# Origin of Increased Reactivity in Rhenium-Mediated Cycloadditions of Tetrazines

**DOI:** 10.1021/acs.joc.1c01564

**Published:** 2021-09-01

**Authors:** Aneta Turlik, K. N. Houk, Dennis Svatunek

**Affiliations:** † Department of Chemistry and Biochemistry, University of California, Los Angeles, California 90095-1569, United States; ‡ Institute of Applied Synthetic Chemistry, TU Wien, 1060 Vienna, Austria

## Abstract

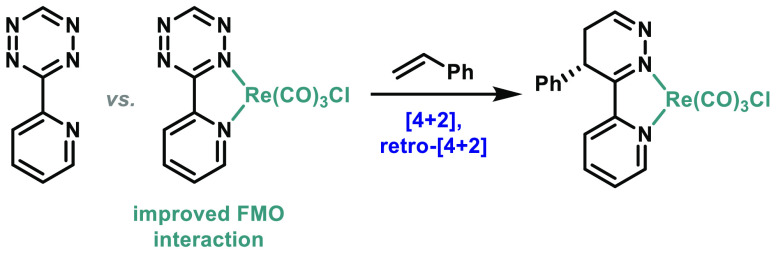

Pyridyl tetrazines coordinated to metals like rhenium have been shown to be more reactive in [4 + 2] cycloadditions than their uncomplexed counterparts. Using density functional theory calculations, we found a more favorable interaction energy caused by stronger orbital interactions as the origin of this increased reactivity. Additionally, the high regioselectivity is due to a greater degree of charge stabilization in the transition state, leading to the major product.

The reaction between 1,2,4,5-tetrazines and alkenes was first described in 1959 by Carboni and Lindsey.^1^ These azines react with olefins in an inverse electron-demand Diels–Alder cycloaddition followed by a cycloreversion with loss of dinitrogen (Figure 1a). The formed dihydropyridazines can then further tautomerize or be oxidized to the corresponding pyridazine.^2^ In 1990, Sauer showed that tetrazines react very rapidly with strained alkenes, such as cyclopropanes and *trans*-cyclooctenes.^3^ This observation led to the introduction of tetrazine ligation as a biocompatible click reaction in 2008.^4,5^ This ligation has since been employed broadly in applications ranging from radiolabeling^6−8^ to material science.^9−11^ A targeted drug delivery system based on this cycloaddition is currently in phase 1 clinical studies.^12,13^

**Figure 1:**
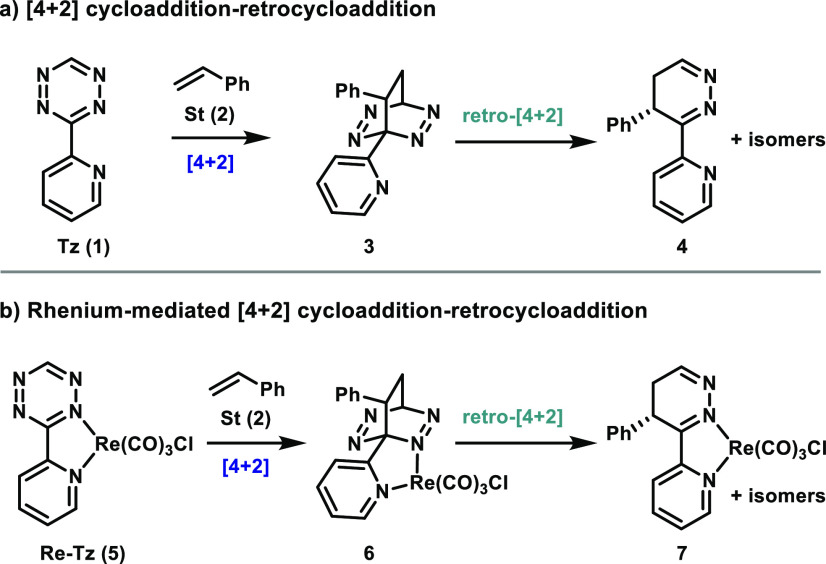
[4 + 2] Cycloadditions of tetrazine **1** (**Tz**) and rhenium-coordinated tetrazine **5** (**Re-Tz**) with styrene (**2**, **St**) leads to the formation of intermediates **3** and **6**, respectively. This is followed by a retro-[4 + 2] cycloaddition to form dihydropyridazines **4** and **7**. The regioisomers in which the Ph group is *ortho* to the pyridyl substituent are shown. Subsequent oxidation of products **4** and **7** can form pyridazines.

Commonly used tetrazines include mono- and disubstituted derivatives with alkyl or aryl groups.^14−17^ Due to the inverse electron-demand nature of the initial rate-limiting [4 + 2] cycloaddition, electron-withdrawing substituents increase reactivity.^18^ 2-Pyridyl- and 2-pyrimidyl-substituted tetrazine derivatives are among the most reactive dienes that can be used for these reactions.^17,19^

2-Pyridyl tetrazines can be used as ligands for metals, as analogues to 2,2′-bipyridine ligands.^20,21^ Recently, Ringenberg and co-workers investigated the click reactivity of a 2-pyridyl tetrazine ligand in a rhenium complex toward vinylferrocene, styrene, and *trans*-cyclooctene (Figure 1b).^22^ This reaction allows for efficient labeling with the metal complex, opening possible applications in the fields of chemical biology and medicine.^23−26^ Interestingly, Ringenberg et al. describe an increase in rate constants of 2 orders of magnitude compared to the reaction of tetrazine itself. The same behavior was observed by the same group for a ruthenium complex.^27^ This acceleration makes these systems particularly interesting as it would allow for labeling at ultralow concentrations. However, the origin of this increased reactivity could not be determined.

Here, we perform a computational analysis using distortion/interaction and energy decomposition analysis to unravel the underlying mechanisms that lead to the observed increase in reactivity. An initial version of this work was deposited in ChemRxiv on July 2, 2021.^28^

## Computational Methods

Density functional theory (DFT) calculations were performed with Gaussian 16 RevA.03.^29^ For each structure, all possible conformers were considered. Geometry optimizations were performed with the B3LYP functional,^30,31^ which was shown to closely match experimental values, augmented with Grimme’s D3 empirical dispersion term,^32−35^ and the SDD basis set for Re and 6-311+G(d,p)^36^ for all other atoms. Dichloromethane solvation was modeled using the SMD solvation model.^37^ Frequency calculations confirmed the optimized structures as minima (zero imaginary frequencies) or transition state structures (one imaginary frequency) on the potential energy surface. A quasi-harmonic correction was applied using the GoodVibes program.^38^ Orbital energies were calculated at the same level of theory in the gas phase.

Distortion/interaction^39^ and energy decomposition analysis^40^ were performed in ADF (2019.304)^41^ with PyFrag 2019^42^ using B3LYP-D3/TZ2P in the gas phase on structures that were optimized with the B3LYP-D3/6-311+G(d,p)/SDD-SMD(DCM) level of theory.

**Figure 2:**
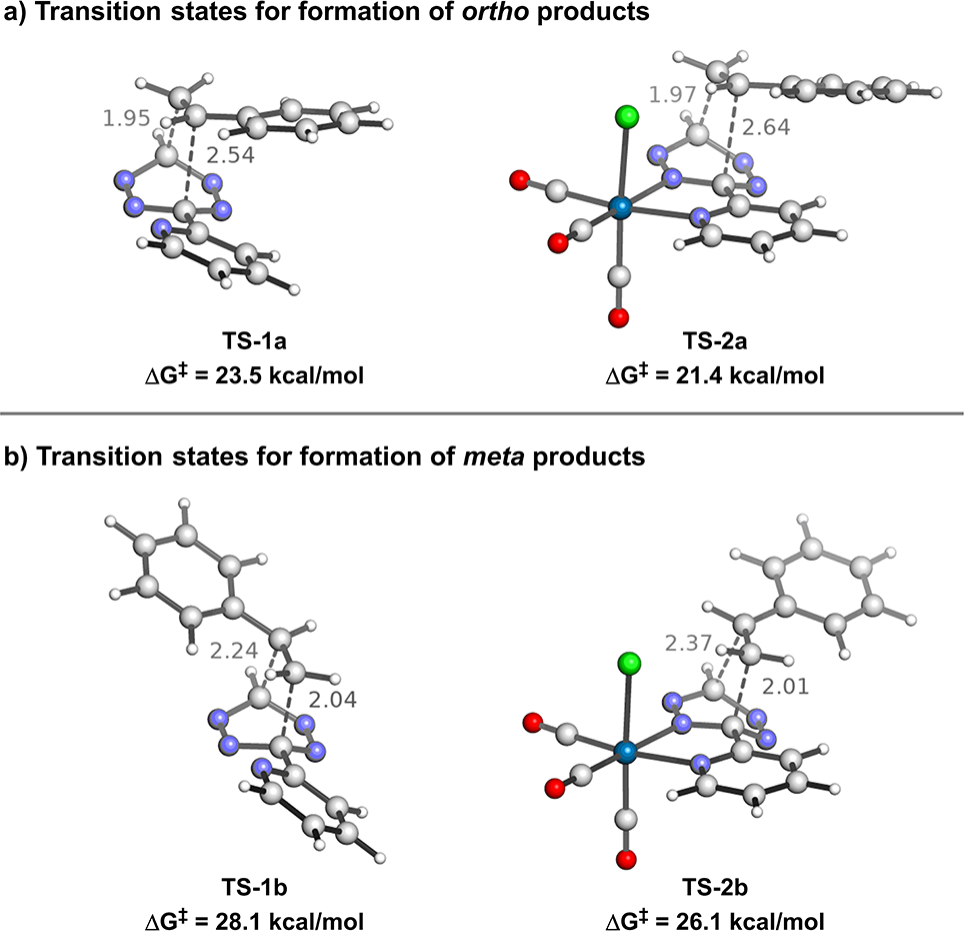
Transition states for the [4 + 2] cycloaddition to form the *ortho* products (a) and the *meta* products (b).

The reactivity of **Tz** (**1**) was first compared to that of **Re-Tz** (**5**) in the reaction with **St** (**2**). In these reactions, the formation of two regioisomers is possible: one in which the phenyl group ends up *ortho* to the pyridyl ring in the final dihydropyridazine (Figure 2a) and one in which these groups are *meta* to each other (Figure 2b). Experimentally, the *ortho* products are the major products observed. The energy barrier for the reaction leading to *ortho* intermediate **3** was calculated to be 23.5 kcal/mol (Figure 2a, **TS-1a**). Coordination of Re reduced this barrier to 21.4 kcal/mol (**TS-2a**) for the formation of *ortho* intermediate **6**. This calculated activation barrier is in excellent agreement with experimental results by Ringenberg and co-workers, whose kinetic experiments correspond to a Δ*G*^⧧^ value of 22.0 kcal/mol. In these reactions, a total of four orientations of addition of **St** to **Tz** are possible; the lowest-energy TSs for the *ortho* and *meta* product are shown in Figure 2. Similarly, the two lowest-energy TSs for the reaction of **St** with **Re-Tz** for formation of the *ortho* and *meta* products out of a possible eight orientations are shown. The other higher-energy transition states and their energy barriers are included in the Supporting Information.

**Figure 3:**
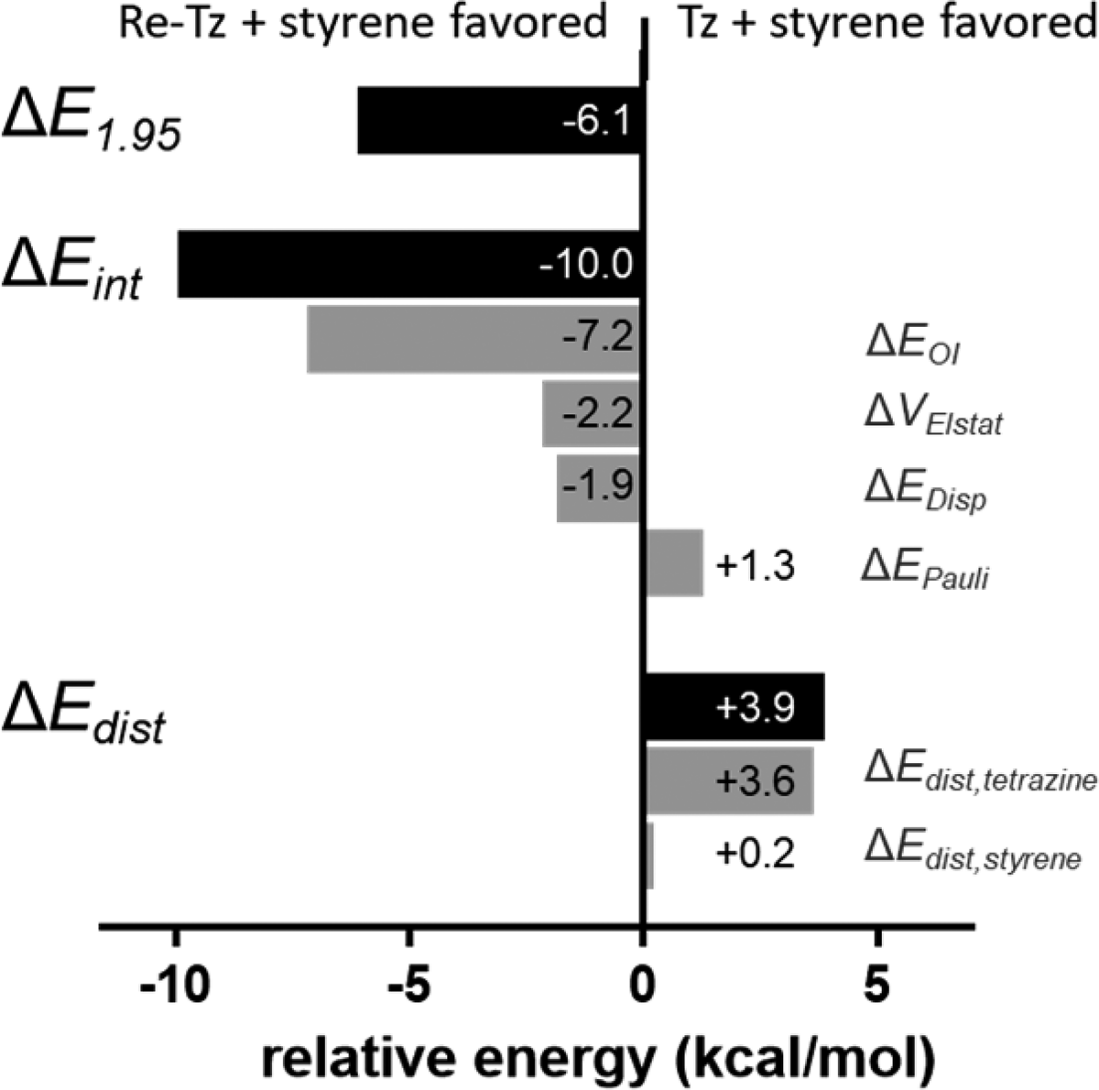
Distortion/interaction and energy decomposition analysis of **TS-1a** and **TS-2a**. Values are shown for **TS-2a** relative to those for **TS-1a**.

In order to determine what causes the lowering of the energy barrier upon coordination of Re, we performed distortion/interaction and energy decomposition analysis (Figure 3). For this analysis, we compared the geometries at the same point along the intrinsic reaction coordinate, in which the shorter bond-forming distance was 1.95 Å. In addition, we performed an analogous analysis of the two structures at the transition states, which can be found in the Supporting Information. At the so-called consistent geometries of 1.95 Å, the electronic energy was lower for the reaction of **Re-Tz** + **St** by 6.1 kcal/mol compared to that of **Tz** + **St**. This difference in electronic energy was decomposed into the interaction (Δ*E*_int_) and distortion (Δ*E*_dist_) components, both of which were decomposed further (Figure 3). Interaction energy was overall favorable for the **Re-Tz** reaction by 10.0 kcal/mol compared to that of the **Tz** reaction. The greatest contribution to this was orbital interactions (Δ*E*_OI_), which were stronger in the **Re-Tz** reaction by 7.2 kcal/mol. Electrostatic interactions (Δ*V*_Elstat_) were also stronger in the reaction with **Re-Tz**, by 2.2 kcal/mol, as were dispersive interactions (Δ*E*_Disp_), by 1.9 kcal/mol. Conversely, Pauli repulsive interactions in the reaction of **Tz** were less unfavorable than that of **Re-Tz** by 1.3 kcal/mol.

Distortion was more unfavorable in the case of the **Re-Tz** reaction by 3.9 kcal/mol. Distortion energies were calculated for the tetrazine/Re-tetrazine and styrene components, and the **Re-Tz** component was shown to be the main contributor to the difference in distortion energies between the two reactions by 3.6 kcal/mol more than the **Tz** component. Distortion in the **St** component was similar in both reactions, with a difference of only 0.2 kcal/mol.

**Figure 4:**
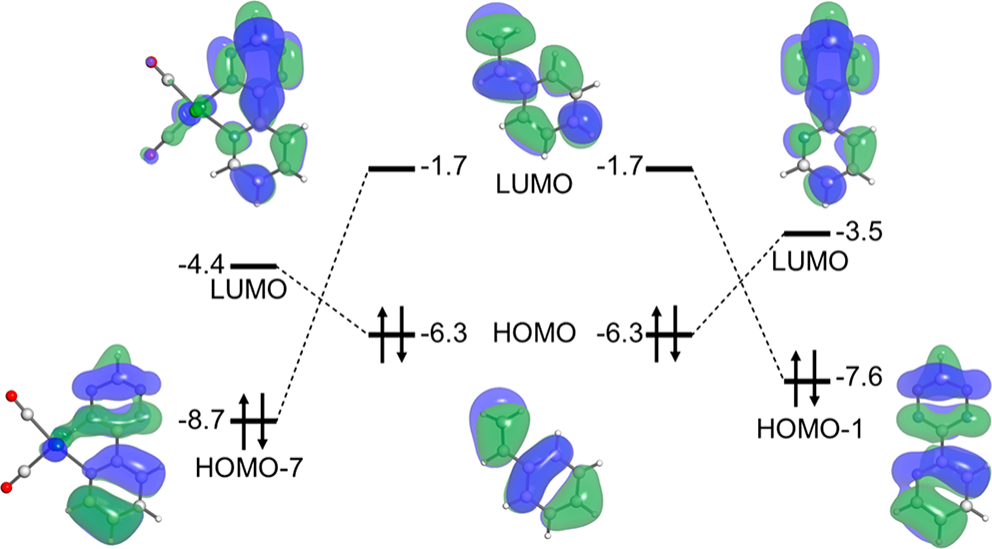
Reacting orbitals and orbital energies of fragments at the consistent geometry where the shorter forming bond is 1.95 Å. Reactions of **Re-Tz** (left) and **Tz** (right) with **St** are shown.

As suggested by energy decomposition of the interaction energies, we performed further analysis of the orbital interactions at play in this reaction. The energies and visualizations of the relevant filled and empty molecular orbitals of the styrene and tetrazine components for the **Tz** and **Re-Tz** structures at the consistent geometry are depicted in Figure 4. The analogous energies for the starting materials and transition states are included in the Supporting Information. In the case of **Re-Tz**, the HOMO is located at the metal center; the Diels–Alder reactive filled orbital is the HOMO-7. As the reaction of tetrazines with dienophiles is an inverse electron-demand Diels–Alder reaction, the orbitals that contribute most to the reactivity are the LUMO of the diene and the HOMO of the dienophile. This can be seen by the HOMO/LUMO gap between the styrene and **Tz**/**Re-Tz**: 2.8 and 1.9 eV, respectively. In contrast, the normal electron-demand orbital gaps are 5.9 and 7.0 eV for **Tz** and **Re-Tz**, respectively (Figure 4). We thus conclude that the enhanced orbital interactions in the **Re-Tz** reaction, which is ultimately the major contributor to enhanced reactivity compared to the **Tz** reaction, originates from a lowered LUMO in the coordinated tetrazine.

We subsequently analyzed the regioselectivity of this reaction. The reaction is highly regioselective, with formation of the *ortho* product favored over formation of the *meta* product. Transition states for formation of the *meta* products are shown in Figure 2b. For the reaction of **Tz** with styrene, the *ortho* TS is lower in energy than the *meta* TS by 4.6 kcal/mol. For the reaction of **Re-Tz** with styrene, this difference is 4.7 kcal/mol. Thus, both systems are highly regioselective. This is unusual for bioorthogonal tetrazine ligation, where poor regioselectivity is often observed.^43^ However, the high degree of regioselectivity in this reaction is not surprising, given the asymmetric electronic and steric nature of both the dienes and dienophile; furthermore, this selectivity has been reported before.^44,45^

**Figure 5:**
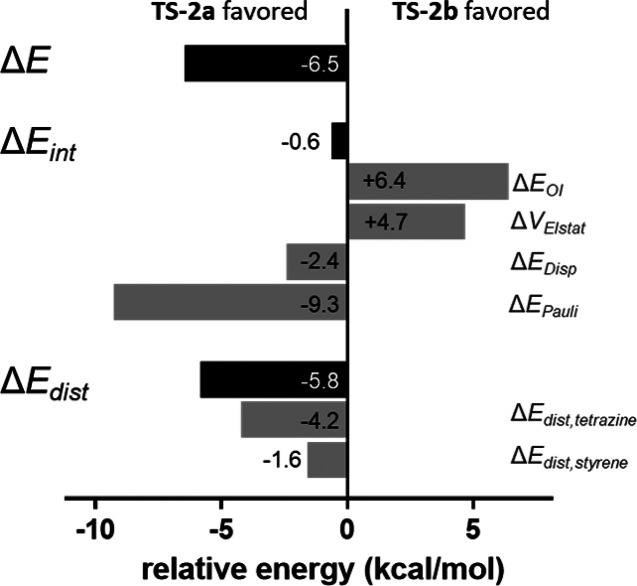
Distortion/interaction and energy decomposition analysis of **TS-2a** and **TS-2b**. Values are shown for **TS-2a** relative to **TS-2b**.

The transition states leading to the favored *ortho* products are more asynchronous than the transition states leading to the *meta* products. In the *ortho* transition state, the more nucleophilic terminal carbon of styrene can interact strongly with the less sterically hindered tetrazine carbon, leading to a short carbon–carbon distance of about 1.96 Å. In addition, charges built up during this interaction can be well-stabilized by the aryl substituent at the *ipso*-carbon, leading to the highly asynchronous and energetically favored approach. In the transition state leading to the *meta* product, the terminal carbon of the styrene also participates in the initially forming bond. In this case, charge buildup on the unsubstituted tetrazine carbon could not be stabilized in a potential asynchronous transition state. This thus leads to a more synchronous transition state that is higher in energy. Comparing **TS-2a** and **TS-2b** using energy decomposition analysis shows that the interaction energy between these two pathways is essentially the same (Figure 5). The highly asynchronous nature of **TS-2a** leads to reduced Pauli repulsion, whereas orbital and electrostatic interactions are less favorable. The HOMO_styrene_–LUMO_tetrazine_ gap is essentially the same in both cases, but the more synchronous **TS-2b** shows an increased HOMO_styrene_–LUMO_tetrazine_ overlap of 0.137 compared to that of **TS-2a** with 0.131. However, distortion energies are considerably lower in **TS-2a** because of the asynchronous bond formation which results in an overall lower barrier.

**Figure 6:**
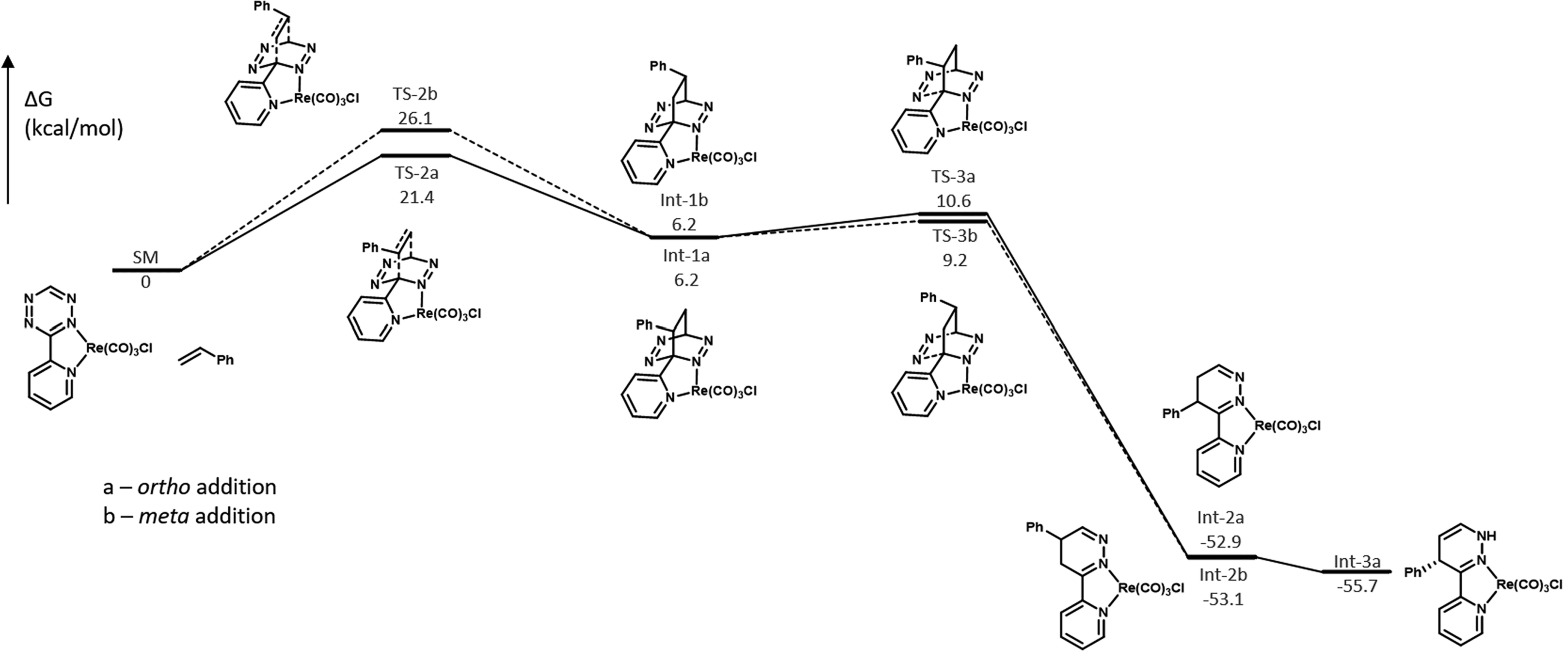
Free energy diagram for formation of *ortho* and *meta* dihydropyridazine products in the reaction of **Re-Tz** with **St**.

For the reaction of **Re-Tz** with **St**, we also studied the reaction profiles for the entire reaction sequence for formation of the *ortho* and *meta* products; these energy profiles are shown in Figure 6. It should be noted that, in both of these pathways, the preferred addition of styrene is from the face that contains the Cl ligand—this mode of addition is favored over addition to the CO face by 0.8 kcal/mol. Both pathways shown in Figure 5 correspond to addition to the Cl face. As discussed previously, the difference in energy barriers between **TS-2a** and **TS-2b** is 4.7 kcal/mol. These transition states lead to the formation of [4 + 2] cycloaddition products **Int-1a** and **Int-1b**, which are both at 6.2 kcal/mol. Subsequent retro-[4 + 2] cycloadditions have low energy barriers at 9.2 and 10.6 kcal/mol relative to starting materials **SM** and are highly exergonic, leading to low-energy products at −52.9 and −53.1 kcal/mol for **Int-2a** and **Int-2b**, respectively. **Int-2a** then preferentially undergoes isomerization to form a double bond between the two distal carbons of the dihydropyridazine (**Int-3a**). **Int-3a**, in which the Ph group is on the same face as the Cl ligand, is 0.2 kcal/mol lower in energy than the epimer in which the Ph group is on the same face as the CO ligand. It is likely that these two structures can reach a thermodynamic equilibrium at this stage through alkene isomerization.

We also studied the possibility of a retro-[4 + 2] cycloaddition occurring with the N_2_ that is coordinated to the Re center. For the *ortho* pathway, the transition state for this reaction was at 22.3 kcal/mol, which is significantly higher than the 9.2 kcal/mol that is observed for retrocycloaddition to expel the distal N_2_. This is reflected in the greater stability and entropy of product **Int-2a** compared to that which would be formed in the case of the other retro-[4 + 2] pathway.

## Conclusion

With the considerable increase in Diels–Alder reactivity, the rhenium-mediated tetrazine ligation adds another interesting tool to the click chemistry toolbox. Using energy decomposition methods, we show that this higher reactivity is mainly due to a lowered LUMO of the tetrazine and therefore increased orbital interactions. These results also suggest that frontier molecular orbital interactions are a good indicator of cycloaddition reactivity of such tetrazine ligands in metal complexes. The high regioselectivity of the reaction with styrene is explained through a better stabilization of charges at the *ipso*-carbon of the monosubstituted tetrazine.

## Supporting Information Available

Energies and geometries of all computed structures, orbital energies at transition state geometry and for starting materials, EDA at transition states (PDF)
Cartesian coordinates of all discussed structures in XYZ file format (ZIP)
