# Supplementary material for: Origin of Increased Reactivity in Rhenium-Mediated Cycloadditions of Tetrazines
Source: J Org Chem. 2021 Sep 1;86(18):13129–33. doi: 10.1021/acs.joc.1c01564 (PMC8453624; doi:10.1021/acs.joc.1c01564)
Supplement: Supplementary file 1 — jo1c01564_si_001.pdf [file jo1c01564_si_001.pdf]

Electronic Supplementary Information

Origin of Increased Reactivity in Rhenium-Mediated Cycloadditions of Tetrazines

Aneta Turlik,† K. N. Houk,† and Dennis Svatunek*,‡

†Department of Chemistry and Biochemistry, University of California, Los Angeles, California 90095-1569, United States

‡Institute of Applied Synthetic Chemistry, TU Wien, 1060 Vienna, Austria

* dennis.svatunek@tuwien.ac.at

Contents

Computational methods 2

Computational data 2

Figure S1. Other, higher-energy transition states for the [4+2] cycloaddition. 2

Table S1: PyFrag results of EDA at the B3LYP-D3/TZ2P level....3

Table S2: Summary of B3LYP-D3/6-311+G(d,p)/SDD-SMD(DCM) energies....4

Table S3: Orbital energies of starting materials and fragments at transition states....4

References....5

Computational methods

Density functional theory (DFT) calculations were performed with Gaussian 16 RevA.03.[1] For each structure, all possible conformers were considered. Geometry optimizations were performed with the B3LYP functional,[2, 3] which was shown to closely match experimental values, augmented with Grimme's D3 empirical dispersion term,[4-7] and the SDD basis set for Re and 6-311+G(d,p)[8] for all other atoms. Dichloromethane solvation was modeled using the SMD solvation model.[9] Frequency calculations confirmed the optimized structures as minima (zero imaginary frequencies) or transition state structures (one imaginary frequency) on the potential energy surface. A quasi-harmonic correction was applied using the GoodVibes program.[10] Orbital energies were calculated at the same level of theory in the gas phase.

Distortion/interaction[11] and energy decomposition analysis[12] were performed in ADF (2019.304)[13] with PyFrag 2019[14] using B3LYP-D3/TZ2P in the gas phase on structures that were optimized with the B3LYP-D3/6-311+G(d,p)/SDD-SMD(DCM) level of theory.

Computational data

Below are the additional high-energy transition states for the initial [4+2] cycloaddition including barriers (Figure SI1), the results of EDA as provided by PyFrag at the transition states and consistent geometry for **TS-1a** and **TS-2a** (Table S1). Table S2 shows the energies of all discussed stationary points and the number of imaginary frequencies (N_{imag}) associated with each structure. Table S3 provides the orbital energies of starting materials and fragments at transition states for **TS-1a and TS-2a**. Geometries are provided as *.xyz files.

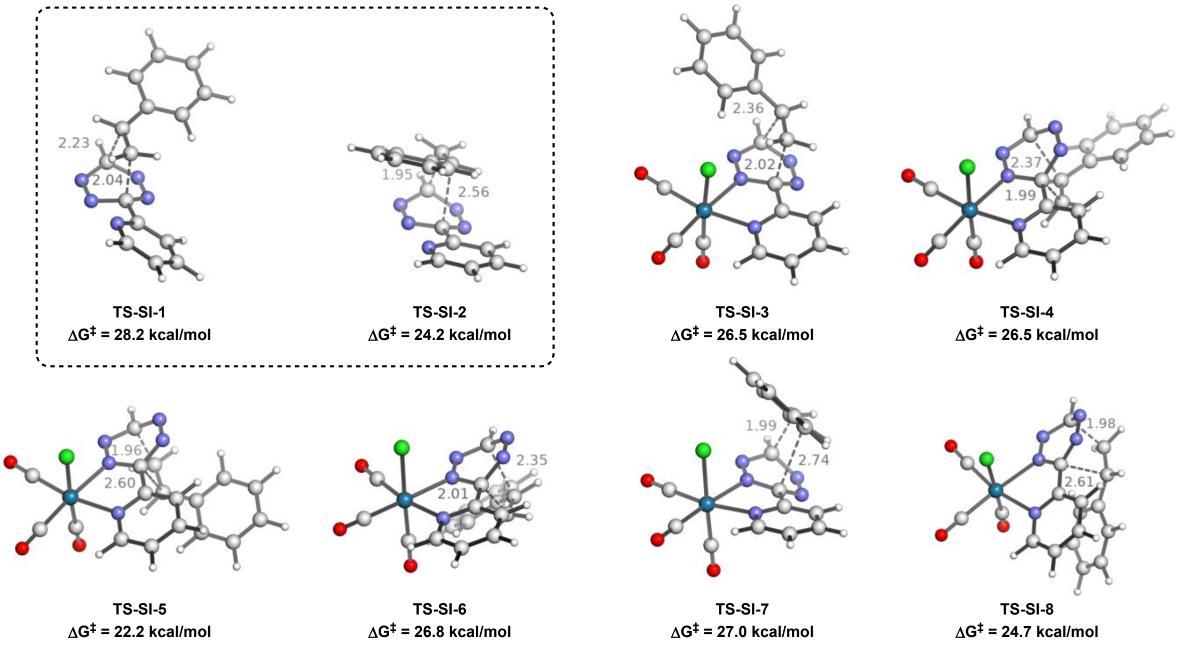

Figure S1. Other, higher-energy transition states for the [4+2] cycloaddition**. TS-SI-1** and **TS-SI-2** are the two other possible transition states without coordination to a Re complex, and **TS-SI-3 to TS-SI-8** are transition states for Re complexes. **TS-SI-1**, **3**, **4**, and **6** lead to formation of *meta* products, and **TS-SI-2**, **5**, **7**, and **8** lead to formation of *ortho* products. **TS-SI-3** and **7** correspond to addition from the face with the Cl ligand, and **TS-SI-4**, **5**, **6**, **8** correspond to addition from the face with the CO ligand.

Table S1: PyFrag results of EDA at the B3LYP-D3/TZ2P level

EDA at the transition state

tetrazine	shorter bond length (Å)	Barrier height (kcal/mol)	ΔE_{int} (kcal/mol)	ΔV_{Elstat} (kcal/mol)	ΔE_{Pauli} (kcal/mol)	ΔE_{OI} (kcal/mol)	ΔE_{disp} (kcal/mol)	ΔE_{dist} (kcal/mol)	$\Delta E_{dist,tetrazine}$ (kcal/mol)	$\Delta E_{dist,styrene}$ (kcal/mol)
**Tz**	1.954	8.317	-17.284	-60.763	124.199	-68.616	-12.104	25.601	17.325	8.276
**Re-Tz**	1.975	2.062	-25.096	-59.739	118.978	-70.367	-13.968	27.158	19.477	7.681

EDA at consistent geometry

	shorter bond length (Å)	Barrier height (kcal/mol)	ΔE_{int} (kcal/mol)	ΔV_{Elstat} (kcal/mol)	ΔE_{Pauli} (kcal/mol)	ΔE_{OI} (kcal/mol)	ΔE_{disp} (kcal/mol)	ΔE_{dist} (kcal/mol)	$\Delta E_{dist,tetrazine}$ (kcal/mol)	$\Delta E_{dist,styrene}$ (kcal/mol)
**Tz**	1.950	8.339	-17.719	-61.408	125.472	-69.68	-12.104	26.058	17.608	8.450
**Re-Tz**	1.950	2.227	-27.689	-63.574	126.74	-76.879	-13.976	29.915	21.237	8.678

Table S2: Summary of B3LYP-D3/6-311+G(d,p)/SDD-SMD(DCM) energies

Structure	N_{imag}	ΔE (au)	ΔH (au)	TΔS (au)	$ΔG_{298}$ (au)	$ΔG_{298}$ (kcal/mol)	$ΔG_{298}$ relative to SM (kcal/mol)
SM: Tz	0	-543.5851	-543.4567	0.04383	-543.5005	-341051.5	
SM: Re-Tz	0	-1422.4632	-1422.2976	0.06481	-1422.3624	-892545.2	
SM: St	0	-309.7505	-309.6104	0.03837	-309.6488	-194307.4	
TS-1b	1	-853.3164	-853.0474	0.05721	-853.1046	-535330.8	28.1
TS-1a	1	-853.3239	-853.0547	0.05713	-853.1118	-535335.3	23.5
TS-2a	1	-1732.2055	-1731.8991	0.07794	-1731.9771	-1086831.2	21.4
TS-2b	1	-1732.1974	-1731.8914	0.07821	-1731.9696	-1086826.5	26.1
Int1a	0	-1732.2352	-1731.9252	0.07613	-1732.0013	-1086846.4	6.2
Int1b	0	-1732.2345	-1731.9246	0.07668	-1732.0012	-1086846.4	6.2
TS-3a	1	-1732.2244	-1731.9171	0.07716	-1731.9942	-1086842.0	10.6
TS-3a'	1	-1732.2052	-1731.8982	0.07742	-1731.9756	-1086830.3	22.3
TS-3b	1	-1732.2268	-1731.9191	0.07748	-1731.9965	-1086843.4	9.2
Int2a	0	-1622.7508	-1622.4529	0.07554	-1622.5284	-1018151.2	-52.9
Int2b	0	-1622.7501	-1622.4526	0.07606	-1622.5286	-1018151.3	-53.1
Int3a	0	-1622.7561	-1622.4572	0.07558	-1622.5328	-1018153.9	-55.7
N_2	0	-109.5543	-109.5454	0.02174	-109.5671	-68754.4	
TS-SI-1	1	-853.3163	-853.0472	0.05716	-853.1043	-535330.6	28.2
TS-SI-2	1	-853.3221	-853.0531	0.05760	-853.1107	-535334.6	24.2
TS-SI-3	1	-1732.1970	-1731.8910	0.07797	-1731.9689	-1086826.1	26.5
TS-SI-4	1	-1732.1969	-1731.8908	0.07815	-1731.9690	-1086826.1	26.5
TS-SI-5	1	-1732.2047	-1731.8982	0.07773	-1731.9759	-1086830.5	22.2
TS-SI-6	1	-1732.1977	-1731.8909	0.07753	-1731.9684	-1086825.8	26.8
TS-SI-7	1	-1732.1952	-1731.8894	0.07876	-1731.9681	-1086825.6	27.0
TS-SI-8	1	-1732.2014	-1731.8941	0.07773	-1731.9718	-1086827.9	24.7

Table S3: Orbital energies of starting materials and fragments at transition states

**Starting materials**					
**Tetrazine**		**Re-Tz**		**Styrene**	
LUMO+1	-2.5	LUMO+1	-3.1	LUMO	-1.3
HOMO-1	-7.5	HOMO-6	-8.1	HOMO	-6.3
**Fragments at transition state TS-1a and TS-2a**					
**Tetrazine**		**Re-Tz**		**Styrene**	
LUMO	-3.3	LUMO	-3.8	LUMO	-1.6
HOMO-1	-7.4	HOMO-6	-8.0	HOMO	-6.2

References

1. Frisch, M. J.; Trucks, G. W.; Schlegel, H. B.; Scuseria, G. E.; Robb, M. A.; Cheeseman, J. R.; Scalmani, G.; Barone, V.; Petersson, G. A.; Nakatsuji, H.; Li, X.; Caricato, M.; Marenich, A. V.; Bloino, J.; Janesko, B. G.; Gomperts, R.; Mennucci, B.; Hratchian, H. P.; Ortiz, J. V.; Izmaylov, A. F.; Sonnenberg, J. L.; Williams; Ding, F.; Lipparini, F.; Egidi, F.; Goings, J.; Peng, B.; Petrone, A.; Henderson, T.; Ranasinghe, D.; Zakrzewski, V. G.; Gao, J.; Rega, N.; Zheng, G.; Liang, W.; Hada, M.; Ehara, M.; Toyota, K.; Fukuda, R.; Hasegawa, J.; Ishida, M.; Nakajima, T.; Honda, Y.; Kitao, O.; Nakai, H.; Vreven, T.; Throssell, K.; Montgomery Jr., J. A.; Peralta, J. E.; Ogliaro, F.; Bearpark, M. J.; Heyd, J. J.; Brothers, E. N.; Kudin, K. N.; Staroverov, V. N.; Keith, T. A.; Kobayashi, R.; Normand, J.; Raghavachari, K.; Rendell, A. P.; Burant, J. C.; Iyengar, S. S.; Tomasi, J.; Cossi, M.; Millam, J. M.; Klene, M.; Adamo, C.; Cammi, R.; Ochterski, J. W.; Martin, R. L.; Morokuma, K.; Farkas, O.; Foresman, J. B.; Fox, D. J. *Gaussian 16 Rev. A.03*, Wallingford, CT, 2016.
2. Vosko, S. H.; Wilk, L.; Nusair, M., Accurate spin-dependent electron liquid correlation energies for local spin density calculations: a critical analysis. *Can. J. Phys.* **1980,** *58* (8), 1200-1211.
3. Head-Gordon, M.; Pople, J. A.; Frisch, M. J., MP2 energy evaluation by direct methods. *Chem. Phys. Lett.* **1988,** *153* (6), 503-506.
4. Grimme, S.; Antony, J.; Ehrlich, S.; Krieg, H., A consistent and accurate ab initio parametrization of density functional dispersion correction (DFT-D) for the 94 elements H-Pu. *J. Chem. Phys.* **2010,** *132* (15), 154104.
5. Lee, C.; Yang, W.; Parr, R. G., Development of the Colle-Salvetti correlation-energy formula into a functional of the electron density. *Phys. Rev. B* **1988,** *37* (2), 785-789.
6. Becke, A. D., Density-functional thermochemistry. III. The role of exact exchange. *J. Chem. Phys.* **1993,** *98* (7), 5648-5652.
7. Stephens, P. J.; Devlin, F. J.; Chabalowski, C. F.; Frisch, M. J., Ab Initio Calculation of Vibrational Absorption and Circular Dichroism Spectra Using Density Functional Force Fields. *J. Phys. Chem.* **2002,** *98* (45), 11623-11627.
8. Krishnan, R.; Binkley, J. S.; Seeger, R.; Pople, J. A., Self-consistent molecular orbital methods. XX. A basis set for correlated wave functions. *J. Chem. Phys.* **1980,** *72* (1), 650-654.
9. Marenich, A. V.; Cramer, C. J.; Truhlar, D. G., Universal solvation model based on solute electron density and on a continuum model of the solvent defined by the bulk dielectric constant and atomic surface tensions. *J. Phys. Chem. B* **2009,** *113* (18), 6378-6396.
10. Luchini, G.; Alegre-Requena, J. V.; Funes-Ardoiz, I.; Paton, R. S., GoodVibes: automated thermochemistry for heterogeneous computational chemistry data. *F1000Research* **2020,** *9*, 291.
11. Bickelhaupt, F. M.; Houk, K. N., Analyzing Reaction Rates with the Distortion/Interaction-Activation Strain Model. *Angew. Chem. Int. Ed.* **2017,** *56* (34), 10070-10086.
12. Bickelhaupt, F. M.; Baerends, E. J., Kohn-Sham Density Functional Theory: Predicting and Understanding Chemistry. In *Rev. Comput. Chem.*, 2000; pp 1-86.
13. te Velde, G.; Bickelhaupt, F. M.; Baerends, E. J.; Fonseca Guerra, C.; van Gisbergen, S. J. A.; Snijders, J. G.; Ziegler, T., Chemistry with ADF. *J. Comput. Chem.* **2001,** *22* (9), 931-967.
14. Sun, X.; Soini, T. M.; Poater, J.; Hamlin, T. A.; Bickelhaupt, F. M., PyFrag 2019-Automating the exploration and analysis of reaction mechanisms. *J. Comput. Chem.* **2019,** *40* (25), 2227-2233.
